# Supernatants derived from chemotherapy-treated cancer cell lines can modify angiogenesis

**DOI:** 10.1038/bjc.2012.13

**Published:** 2012-01-31

**Authors:** W M Liu, J L Dennis, A M Gravett, C Chanthirakumar, E Kaminska, G Coulton, D W Fowler, M Bodman-Smith, A G Dalgleish

**Affiliations:** 1 Department of Oncology, Division of Clinical Sciences, St George's, University of London, 2nd Floor, Jenner Wing, London SW17 0RE, UK; 2 Medical Biomics Centre, Division of Basic Molecular Sciences, St George's, University of London, London, UK.

**Keywords:** tumour-supernatants, chemotherapy, immunotherapy, angiogenesis, immune-modulation, microvesicles

## Abstract

**Background:**

There is evidence that tumours produce substances such as cytokines and microvesicular bodies bearing bioactive molecules, which support the carcinogenic process. Furthermore, chemotherapy has also been shown to modify these exudates and in doing so, neutralise their tumourigenic influence.

**Methods:**

In the current study, we have investigated the effect of chemotherapy agents on modifying the cytokine profile and microvesicular cargo of supernatants derived from cancer cell lines. In addition, we have explored the effect of these tumour-derived supernatants on angiogenesis, and how chemotherapy can alter the supernatants rendering them less pro-angiogenic.

**Results:**

Herein, we show that supernatants contain a rich cocktail of cytokines, a number of which are potent modulators of angiogenesis. They also contain microvesicular bodies containing RNA transcripts that code for proteins involved in transcription, immune modulation and angiogenesis. These supernatants altered intracellular signalling molecules in endothelial cells and significantly enhanced their tubulogenic character; however, this was severely compromised when supernatants from tumours treated with chemotherapy was used instead.

**Conclusion:**

This study suggests tumour exudates and bioactive material from tumours can influence cellular functions, and that treatment with some chemotherapy can serve to negate these pro-tumourigenic processes.

We have previously shown that some chemotherapies are able to affect immune modulation (Liu *et al*, 2010). This is manifest in two ways, first chemotherapy can upregulate class I human leucocyte antigen expression on tumours directly, which leads to an improved adaptive response. Second, an indirect effect of chemotherapy is possible as supernatant derived from tumours treated with standard drugs such as oxaliplatin (OXP) or gemcitabine can enhance the function of dendritic cells (DCs). Specifically, DCs exposed to these supernatants have an enhanced maturation phenotype, which in turn increases the activation and proliferation of T-lymphocytes, and ultimately the cytolytic ability of these effector cells. The effects on DCs were apparent at the level of gene expression, with the supernatants being capable of modifying the expressions of DC markers such as CCR7, CD80 and CD86 (Liu *et al*, 2012).

It appears that chemotherapy can hijack tumours resulting in their producing substances that are anti-tumour in character. Moreover, as tumours seem to intrinsically produce these substances, chemotherapy may actually serve to negate/neutralise the action of tumour-derived supernatants. Indeed, the presence of a bridge of communication between tumours and the host microenvironment suggests its role in the tumourigenic process (Joyce and Pollard, 2009). Communication can be direct through tumour-to-stroma contact, or via an indirect mechanism such as supernatants drawn from tumours. These supernatants invariably reflect the cellular and biochemical makeup of the tumour, and can exhibit a repertoire of cytokines (Dranoff, 2004). Their components appear to support tumourigenesis, but the particular roles they have in the process are unclear. However, we have shown that tumour-derived cytokines can support angiogenesis (Liu *et al*, 2009), and directly maintain an immunosuppressive microenvironment conducive to cancer development (Dalgleish and O’Byrne, 2002).

Supernatants can also include cellular fragments, apoptotic bodies and other plasma membrane-derived vesicles. These microvesicular structures have been detected in a number of biological fluids including blood and ascitic fluid, and are thought to be involved in communication between cells, including cross talk between tumour cells and host (Thery *et al*, 2002). In a similar way to cytokines, microvesicles derived from some tumours can have a part in suppressing immune responses directed towards the cancer, as well as modifying the tumour microenvironment to support tumourigenesis. Although the exact mechanism by which this extracellular communication is achieved is unknown, RNA and microRNA material have been found within the vesicles as well as adhered to these microparticles, thus rendering them translatable protagonists (Valadi *et al*, 2007; Pegtel *et al*, 2010). The resulting effects have included increased metastatic potential and modulation of angiogenesis (Hood *et al*, 2011; Taverna *et al*, 2011).

As part of our ongoing research studies, we have examined more closely the role of supernatants derived from cancer cell lines before and after treatment with chemotherapy in modifying the interaction between these cells and the host microenvironment. We have worked on the hypothesis that supernatants contain bioactive substances derived from tumours, which modify the tumour-host relationship in a way that is favourable for tumourigenesis. We have as part of this approach, analysed the cytokine profiles of supernatants, in addition to employing gene microarray analysis to identify key transcripts present in the microvesicles that may be involved in this oncogenic process. The effect that common chemotherapy has on the constitution of these supernatants has also been assessed.

## Materials and Methods

### Cell culture

The human cancer cell lines A549 (lung), HCT116 (colon), MCF7 (breast) and human umbilical vein endothelial cells (HUVECs) were obtained from the Cancer Research UK Cell Production Laboratories (Potters Bar, UK). All cells were maintained in culture medium supplemented with 10% (v/v) foetal bovine serum (FBS), 2 mM L-glutamine and 1% penicillin/streptomycin (basal culture medium). All cell lines were incubated in a humidified atmosphere with 5% CO_2_ in air at 37 °C, and cancer cell lines were discarded when the passage number exceeded 15.

### Reagents

Cyclophosphamide (CPM: Sigma Ltd., Dorset, UK) and OXP (Sigma) were dissolved in phosphate buffered saline (PBS) to create 10 mM stock solutions that were maintained at −20 °C for no longer than 4 weeks. All controls used in our studies involved treatment with equal amounts of PBS.

### Generating tumour-derived supernatants

Supernatants were decanted from these cultures as described previously (Liu *et al*, 2012). Briefly, exhausted media (supernatants) were obtained from cells either cultured alone, with 100 *μ*M CPM or with 1 *μ*M OXP for 72 h. These concentrations were the approximate IC25 s for the drugs as reported previously (Liu *et al*, 2010). All supernatants were stored at −20 °C, and freeze–thaw cycles kept to a minimum by aliquoting.

### Cytokine analysis of supernatants

The profile of 46 cytokines and analytes associated with inflammation were determined in each of the supernatants by a proprietary multiplex immunoassay solution (Rules Based Medicine Inc., Austin, TX, USA). In addition, the levels of VEGF were assayed using an ELISA kit according to the manufacturer's instructions (R&D Systems, Oxford, UK).

### Isolation of microvesicles from supernatants

Small cellular vesicles and microparticles were isolated by sequential centrifugation at 4 °C using increasing forces. These were loosely defined as microvesicles. Culture supernatants were initially centrifuged at 300 **g** for 10 min, and then transferred to a fresh tube for a second spin at 2000 **g** for 10 min. These steps allowed for the separation of live and dead cells, respectively. Supernatants were then centrifuged for a third time at 10 000 **g** for 30 min as a way of removing cellular debris. The supernatants were then transferred to fresh tubes, and ultra-centrifuged at 100 000 **g** for 70 min, before transferring the supernatants to a fresh tube. These samples were defined as the ‘cytokine fraction’. The remaining pellets were then re-suspended in PBS. A final spin at 100 000 **g** for 70 min was performed before supernatants were aspirated, yielding pellets of microvesicles that were stored at −80 °C until required.

### RNA extraction

RNA was extracted from microvesicles collected from the supernatants of A549 cells cultured alone, with CPM or with OXP. RNA was purified by Trizol, followed by precipitation with iso-propanol. The RNA pellet was washed in 70% (v/v) ethanol, air dried, re-suspended in RNase-free water and stored at −80 °C. RNA concentration and purity were measured using a NanoDrop ND 1000 spectrophotometer (NanoDrop Technologies, Wilmington, DE, USA), and RNA integrity was determined by an Agilent 2100 Bioanalyser (Agilent Technologies UK Ltd., Stockport, UK) using RNA 6000 Nano LabChips (Agilent). RNA integrity was expressed in terms of an RNA integrity number as determined by the proprietary software and only those with values of >9.0 were progressed.

### Illumina microarrays

Biotinylated cRNA was generated from 100 ng total RNA using the Illumina Total Prep RNA Amplification Kit (Applied Biosystems, Warrington, UK) according to manufacturer's instructions. The concentration and purity of resultant cRNA was determined using the NanoDrop ND 1000 spectrophotometer (NanoDrop Technologies). Equal amounts (750 ng) of cRNA were hybridised to the Illumina human HT12-v3 arrays (Illumina UK, Saffron Walden, UK) for 18 h and subsequently processed according to manufacturer's instructions before scanning on an Illumina Bead Array Reader (Illumina UK). The image data were processed using default values in Genome Studio v2009.1 (Illumina, UK), before loading onto Gene Spring v9.0 (Agilent Technologies UK Ltd.) for data normalisation and filtering. Analyses were performed using gene ontology databases within Pathway Studio v7.1 (Ariadne Genomics, Rockville, MD, USA). A greater than 0.25-fold change was used as our cutoff magnitude for gene list compositions by using Excel software (Microsoft UK, Reading, UK).

### Tubule-formation assay

The vasculogenic natures of supernatants, microvesicles and cytokine fractions were assessed by their ability to induce the reorganisation on HUVECs into primitive tubules. A layer (150 *μ*l) of growth factor reduced Matrigel matrix was allowed to set in six-well plates for 30 min, before layering on HUVECs (5 × 10^5^ in medium). This medium was either (i) complete supernatants (500 *μ*l) derived from A549 cells cultured with chemotherapy; or (ii) isolated microvesicles re-suspended at a protein concentration of 10 *μ*g per 500 *μ*l in PBS; or (iii) cytokine fraction (500 *μ*l). Samples were incubated in a humidified atmosphere with 5% CO_2_ in air at 37 °C for 16 h, before visualising tubules under light microscopy. Tubule formation was quantified by using the programme AngioQuant (http://www.cs.tut.fi/sgn/csb/angioquant/).

### Immunoblotting analysis

Cells were harvested and total cellular protein was solubilised in lysis buffer (New England Biolabs, Hitchin, UK) and resolved by tris-glycine electrophoresis using a 4–12% bis-tris gradient-gel. Following transfer of proteins to 0.45 *μ*m nitrocellulose membranes, blocking was performed in 5% (w/v) non-fat milk in TTBS (0.5% (v/v) Tween-20 in tris buffered saline (50 mM Tris base with 150 mM NaCl; pH 7.6)). Primary antibody probing was performed with anti-AKT, anti-phospho AKT, anti-ERK and anti-phospho ERK. All primary antibodies were obtained from New England Biolabs and used at a dilution of 1 : 1000, unless stated otherwise. Anti-*β* actin was used as a loading control (1 : 2000 – New England Biolabs). Following three washing steps in TTBS, horseradish peroxidase-conjugated anti-species IgG_1_ was used as the secondary antibody (Amersham Biosciences Ltd., Little Chalfont, UK). Bands were visualised by the ECL-plus detection system (Amersham).

## Results

### Tumour-derived supernatants contain cytokines

**Figure 1:**
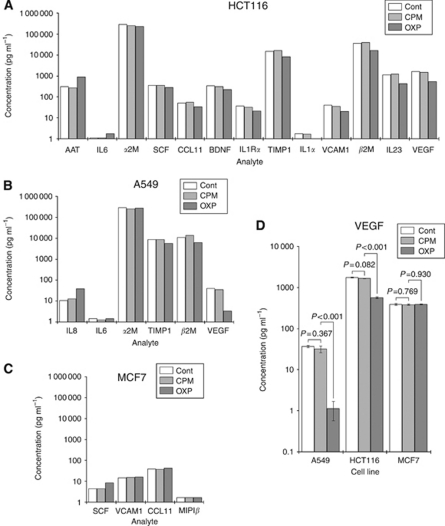
Effects of treatment on cytokine profile. A549, HCT116 and MCF7 cells were cultured with PBS (Cont), CPM or OXP for 72 h before assessing the levels of 46 analytes by a multiplex immunoassay (**A–C**). The amounts of VEGF in the supernatants were also specifically assessed by ELISA (**D**). Data for VEGF represent the mean and s.d.s of three separate experiments, and *P*-values are from paired *t*-tests.

The profiles of cytokines in the supernatants derived from cell lines were assessed by a multiplex immunoassay, where 46 analytes associated with inflammation were quantified. The effect of treating cells with equi-active concentrations of CPM or OXP on cytokine profiles was also established. Only those analytes that were detectable are presented. Results indicated that the supernatants contained variable amounts of cytokines, which were different in the cell lines studies (Figures 1A–C). Specifically, HCT116 cells produced supernatants that consisted of 13 cytokines, compared with 6 and 4 for A549 and MCF7, respectively; and there were no cytokines that were common to all three tumours. Culturing cells with CPM did not affect the levels of these cytokines in the cells; however, there was a divergence in the levels of the cytokines after treatment with OXP. Cytokines were thus ordered according to the difference in amounts between untreated and OXP-treated samples, and VEGF was seen to be altered to the greatest extent. The amount of VEGF in the supernatants were then re-assessed by ELISA, and results showed that they were significantly decreased after treatment with OXP in A549 (1.1±0.55 pg ml^−1^ *vs* 36±2.4 pg ml^−1^ in untreated controls) and in HCT116 (571±29 pg ml^−1^ *vs* 1741±48 pg ml^−1^ in untreated controls) (Both *P*<0.001 Figure 1D).

### Tumour-derived supernatants can support vasculogenesis

**Figure 2:**
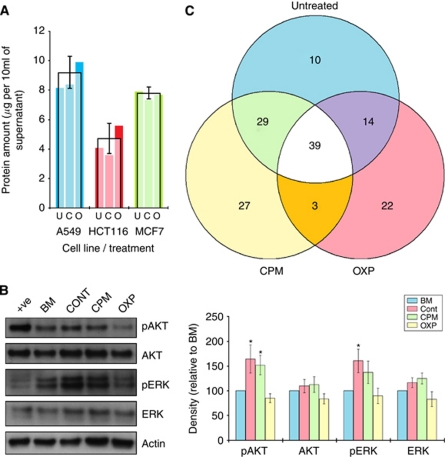
Microvesicles are altered by treatment. A549, HCT116 and MCF7 cells were cultured with PBS (U), CPM (C) or OXP (O) for 72 h before microvesicles were extracted. The protein content of these particles from 10 ml of supernatant was assessed by BCA testing (**A**).The effects of supernatants on the ERK and PI3-K in HUVECs were also examined by western blotting (**B**). +ve refers to effects of endothelial cell medium. Venn analysis of the genes called present in the microvesicles following treatments revealed a large number of genes was common to each condition (**C**). ^*^P<0.05 by paired *t*-tests. All supernatant samples naturally included FBS as part of the exhausted media from which they were derived, and changes in the microvesicle portion would account for any microvesicles derived from FBS.

We next tested the vasculogenic nature of supernatants by culturing them with HUVECs and assessing the extent to which they re-arranged into primitive tubules when grown upon Matrigel. The supernatants were deconstructed by differential ultra-centrifugation into microvesicular and cytokine fractions. The protein contents of the microvesicular fractions were assessed by a proprietary bicinchoninic acid assay kit (Fisher Scientific UK Ltd., Loughborough, UK) (Figure 2A), and then used at a concentration of 10 *μ*g per 500 *μ*l in PBS. There was thus a total of three samples assessed: (1) complete supernatant, (2) microvesicular fraction and (3) cytokine fraction.

**Figure 3:**
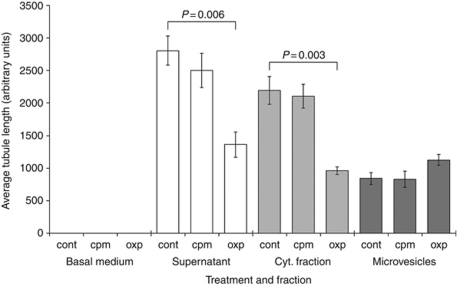
The effect of supernatants on tubulogenesis. HUVEC were cultured with the supernatants derived from A549 cells, and the potential of HUVECs cells to form primitive tube-networks was assessed upon growth factor reduced Matrigel. Tube formation was seen when HUVECs were cultured with CONT-supernatants; however, this was significantly reduced when OXP-supernatants were used. Standard culture medium (basal medium) for tumours induced no tube-formation, as did basal medium spiked with CPM or OXP. Supernatants were then deconstructed into a microvesicular fraction and a cytokine fraction (cyt. fraction), and the effects that these components had on tubulogenesis also assessed. AngioQuant software (see text) was employed to enumerate tube formation, and the values represent the means and s.d.s of four separate samples.

Results showed that culturing HUVECs with standard culture medium or with basal medium spiked with 100 *μ*M of CPM or 1 *μ*M of OXP for 72 h, resulted in no tube formation (Figure 3). However, culturing with CONT-supernatant significantly increased the capacity of HUVECs to form tube-like structures. This was enumerated by measuring the total lengths of tubule-complexes by using the dedicated image analysis tool AngioQuant, which reported an increase from 0 to 2806±225 a.u. (Figure 3). Furthermore, all cells were confirmed viable by trypan blue exclusion analysis. CPM-supernatants also resulted in a similar extent of tubulogenesis (paired *t*-test *vs* controls: *P*=0.161); however, OXP-supernatant significantly reduced the level of tube formation (1362±192 *vs* 2806±225 in CONT-supernatant; *P*=0.006). The trend in tubulogenesis following stimulation with cytokine fractions was similar to that seen with complete supernatants, in that the level of network formation was high in CONT and CPM samples, but significantly reduced in OXP samples (961±57 *vs* 2193±211; *P*=0.003). HUVECs stimulated with microvesicles generally resulted in fewer tubes as compared with the number seen with complete supernatants. Specifically, although microvesicles from untreated tumours significantly increased tubule formation, the amount was much lower than that induced by complete medium (842±91 *vs* 2806±225; *P*<0.001) (Figure 3). There was no difference in the tubulogenic nature of microvesicles from CPM-treated and untreated tumours (*P*=0.886). Conversely, those from OXP-treated tumours had slightly increased tubulogenic capability, which did not reach significance (*P*=0.064) (Figure 3).

### Tumour-derived supernatants alter the expressions of AKT and ERK

To investigate whether the changes in vasculogenesis were linked with modifications to key intracellular signalling pathways, whole-cell lysates were obtained from HUVECs cultured with the supernatants and the levels of AKT and ERK assessed by immunoblotting. This panel was chosen as they broadly represented key proteins involved in modifying angiogenesis and neovascularisation. The intention was to employ this approach to identify whether or not a change in intracellular signalling was involved in triggering the phenotypic changes, which could ultimately provide putative targets against which therapies could be adapted. Results highlighted increases in active AKT and ERK proteins following culture with CONT-supernatants and CPM-supernatants (Figure 2B). However, these increases were not seen in HUVECs cultured with OXP-supernatant, and their levels remained virtually unchanged compared with HUVECs in basal medium (Figure 2B).

### Sample-descriptions, microarray data quality control, filtering and pre-processing

There were two independent sets of microvesicles harvested from A549 tumour cells in separate experiments, and each set comprised of three treatment conditions: (i) untreated; (ii) CPM-treated and (iii) OXP-treated, from which RNA was extracted. The qualities of the RNA from each of the samples were confirmed by Illumina software (Illumina), and all controls (hybridisations, negative, spike-ins, etc) were within the guidelines as specified by the manufacturer. All pre-normalised intensity signals from each probe were collated and those genes whose expressions were below the baseline/background value of 60 were defined as absent. Additionally, those probes with no names or those designated ‘predicted genes’ were also excluded. This filtering process trimmed the number of genes from 45 599 to 28 557. Differences in the magnitudes of gene expressions between any of the treatment groups were then analysed using Excel software.

### Comparing the transcriptomes of microvesicles

An initial survey of the genes showed there to be a very small number present in the microvesicles. For instance, there were just 92 hits from a possible 28 557 genes (0.32%) in the microvesicles from the supernatants of untreated tumour cells. Full gene lists are available at GeneExpress (www.ebi.ac.uk – accession number E-TABM-1201). A large proportion of these genes possessed similar functions, so gene ontological analysis using Pathway Studio was performed on this list. Categories were then defined and ranked in order of the percentage of genes found. The leading categories were regulation of transcription (15% (12/92)), and genes for membrane components (27% (25/92)) (Table 1). The expressions of these 92 genes were then established in the microvesicles harvested from tumours cultured with CPM or OXP (Table 1). Venn analysis of all the transcripts called present in the microvesicles from any of treatments showed a proportion of them was common (Figure 2C). There were overlapping genes unique to CPM or OXP with the untreated sample (28 and 14, respectively), but few between CPM and OXP (3) (Figure 2C).

In an attempt to understand the genetic backdrop of the general observation that the vasculogenic action of microvesicles were not different between CPM and untreated samples but different in OXP-treatment, a second list of genes were created that complied with the criteria that expressions be (i) <10% different between untreated and CPM-treated, and (ii) >1-fold change in either direction between untreated and OXP-treated (Table 2). Furthermore, this approach was also in accordance with our previous published data that indicated CPM generally affected cells to a lesser degree than OXP (Liu *et al*, 2010, 2012). Analysis revealed just 13 genes (0.046%) conformed to these criteria, and that a number of them (*il18*, *lilrb1*, *krit1*, *abca4* and *map1a*) were associated with angiogenesis and neovascularisation.

## Discussion

This study was initiated in response to our earlier reports defining a novel potential immune-stimulatory feature of some common chemotherapy agents. In these studies, we showed an enhancement to cell-mediated immune responses by supernatants derived from tumour cells, and that these were affected by drug treatments (Liu *et al*, 2010). Therefore, the aim of the current study was to explore the biological nature of these supernatants. To this end, we deconstructed the supernatants into cytokine and microvesicular fractions, and employed molecular biological techniques to establish the effect on cellular processes of the individual contributions of these parts. Our results showed that the supernatants were rich in cytokines with the capacity to alter angiogenesis, as well as containing microvesicles loaded with RNA material. These supernatants altered intracellular signalling, reorganised the phenotype of HUVECs, and thus modified the process of tubulogenesis. Importantly, our data also supported the idea that some chemotherapy could negate these effects, and hence minimise the impact of these neoplastic events.

Cancer cells produce a heterogeneous mixture of material that has been loosely referred to as debris, apoptotic fragments or microvesicular matter. Rather than being haphazard and inert in nature, they have been shown to be organised into vesicles filled with bioactive material (Thery *et al*, 2002). Their presence in biological fluids such as blood, urine and ascites supports their role in cell-to-cell communication, and suggests an involvement in the carcinogenic process (Zhang and Grizzle, 2011). The overlapping association of these microvesicles with soluble factors such as cytokines, which together are constantly exuded by tumours generates a potent mixture that is capable of transmitting developmental cues between tumour and host cells. It would be valuable to identify the key elements in the supernatants as this would assist in developing new therapeutic methods that would negate or minimise their impacts. Not only that, this would also help us understand our earlier data. For that reason, we assessed the cytokine fraction of the supernatants by using a multiplex immunoassay that simultaneously quantified 46 analytes related to inflammation. Results showed that the cytokines identified in the supernatants were varied and tumour specific. The specific roles and impacts of these analytes were not assessed; however, it appeared that a number of these were associated with angiogenesis. In particular, there were a number of analytes with strong links to neo-vascularisation that were detected in the supernatants of untreated HCT116 cells, and of these, we focused primarily on VEGF, the cytokine that was altered the greatest.

In addition to our initial multi-analyte profiling approach, we also separated the microvesicular fraction from the soluble and debris fractions of the supernatants by differential ultra-centrifugation (Thery *et al*, 2006), and assessed the genetic cargo of these membrane bound particles by microarray analysis. Technically, this population of microvesicles would have been mixed with those shed by FBS (Heijnen *et al*, 1999). However, as FBS was present in all our supernatants, any changes to their profile would be automatically factored in. Our initial intention was to use this methodology as a way of first, establishing if any the presence of RNA transcripts; and second, to identify those that were found. We intentionally did not treat these microvesicles with RNAse to remove surface RNA, as physiologically, these may also have a putative role in communication. Additionally, it was not our focus to discriminate the individual components of the microvesicles (Simpson *et al*, 2008), and thus treated them as one entity. Results of the gene expression analysis revealed just 92 genes out of a possible 28 557 genes (0.32%) were found in the microvesicles. A number of these genes were primarily associated with transcription and immune function, which reinforced our initial studies that suggested tumour-derived supernatants were exploitable targets for chemotherapy with regards to enhancing immunosurveillance and immune function (Liu *et al*, 2010, 2012). Interestingly, the results of this analysis also identified a cohort of genes specifically involved in angiogenesis was also represented in the microvesicles.

To address the suggestion that supernatants were able to influence angiogenesis, we next assessed the ability of tumour supernatants to re-organise HUVECs into primitive tube-structures on Matrigel. We first demonstrated an increased active state of intracellular molecules ERK and AKT in HUVECs, which are important protagonists intimately associated with the angiogenesis (Carmeliet and Jain, 2011), following treatment with supernatants from tumours. The phenotypic consequence of this was significant increases in tube formation by HUVECs. This is not only seen in complete supernatants, but also seen when HUVECs were stimulated specifically with the cytokine fraction of deconstructed supernatant. This was a possible reflection of the pro-angiogenic factors detected by the immunoassays. Moreover, a primitive network of tubes was also seen after treatment with microvesicles, albeit to a much lower extent. This effect has been reported recently in chronic myeloid leukaemia (Taverna *et al*, 2011). Our data also suggested that treating tumour cells with OXP may have resulted in microvesicles that were more angiogenic in nature, as there was a modest increase in tubulogenesis following culture with these microvesicles. Although this increase was not statistically significant (1124±84 *vs* 842±91 using untreated tumour microvesicles; *P*=0.064), we are currently exploring the fascinating notion that chemotherapy may modify the microvesicular output of tumours, which may influence the tumour–host relationship. Equally, we are also investigating the fundamental nature of the microvesicular fraction, which may be sufficiently altered by different chemotherapies to cause differences in macroscopic effects. For instance, OXP may cause there to be being more apoptosomes within the microvesicular portion of supernatants.

Thus, it appears that tumours are capable of depositing into the microenvironment factors that benefit growth, which may represent a mode by which tumours promote metastasis. For this reason, antagonising the action of these factors would be therapeutically attractive. Indeed, we have previously shown that negating the effects of cytokines exuded by peripheral blood mononuclear cells can drastically potentiate cytolytic T-cell activity (Liu *et al*, 2011). Therefore, we assessed the impact that culturing tumour cells with CPM and OXP would have on the composition of supernatants. Our earlier studies had already defined a benefit to immune function of these supernatants (Liu *et al*, 2010, 2012), and although the current study was tasked to explore this further, we instead focused on the effects that chemotherapy may have on the angiogenesis-promoting character of tumour supernatants, as well dissecting the roles that the individual parts of the supernatant may possess. Our results showed that treating tumour cells with OXP, altered the profile of the supernatant exuded by the tumours to render them less angiogenic in nature. Parenthetically, treating tumour cells with an equi-active concentration of CPM did not affect the angiogenic quality of the supernatant, which was manifest as a lack of an impact on tubulogenic action in HUVECs. This highlights the intrinsic differences in the mechanisms of action for these two drugs that produced such divergent effects; indeed, we have shown previously supernatants from A549 cells have distinct immune stimulatory activity (Liu *et al*, 2012), which can only be a reflection of the way that these agents work and the intracellular pathways that are affected.

In summary, the current study initially tasked to explicate results of our earlier studies showing an immunity-enhancing effect of common chemotherapy has instead highlighted an effect of the chemotherapy on angiogenesis. The effect of chemotherapy was indirect, and caused by modifications to supernatants produced by tumours following treatment with the drugs. The idea that supernatants produced by tumours that are fundamentally supportive of tumour growth can be disrupted by chemotherapy, as well as their being capable of enhancing immune responses, suggests substances are capable of being transmitted from cell to cell. This form of communication that in part regulates tumour development thus forms a new therapeutic target, which presents an interesting avenue for research. For instance, using blocking antibodies to negate the actions of the cytokines within the supernatants may prove fruitful, and suggests a benefit of combining agents with overlapping functions to counter the impact of tumour exudates. This is another particular area of research that we are pursuing.

## Figures and Tables

**Table 1:** Top-40 genes present in tumour microvesicles

**Gene expression**					**Gene expression**				
**CON**	**CPM**	**OXP**	**Symbol**	**Notes**	**CON**	**CPM**	**OXP**	**Symbol**	**Notes**
3193	3193	3193	LAIR1	Leukocyte immunoglobulin-like receptor 1	438	132	910	SALL3	
2720	2720	2720	IMAA	Pseudogene	421	417	383	CDAN1	Congenital dyserythropoietic anaemia type 1
2146	2084	2146	CLUAP1	Clusterin-associated protein 1	395	371	194	GABPB2	GA-binding protein transcription factor *β* subunit 2
2022	2084	2022	F2R	Thrombin receptor	387	438	362	SHCBP1	Associated with intracellular signalling
1464	1464	1387	C19orf31		383	352	200	SLC44A4	Solute carrier
1310	1310	1172	FARSLB	Phenylalanyl-tRNA synthetase, *β* subunit	367	464	328	MSH3	Component of the DNA mismatch repair system
1172	1172	1387	NAG18		366	444	217	MGC16703	
985	971	965	ITIH5	Inter-*α* inhibitor H5	364	362	188	C21orf55	
971	968	955	ROCK2	Rho kinase 2	336	309	66	PNPT1	Polynucleotide phosphorylase 1
955	971	874	LILRB3	Leukocyte immunoglobulin-like receptor 3	330	304	76	IL18	Interleukin 18
929	929	955	ORC6 L	Origin recognition complex subunit 6	325	327	51	RPL7L1	Ribosomal protein
743	740	683	LOC400721		318	103	659	FGA	Processes fibrin
708	740	616	KIAA0101	Associated with proliferating cell nuclear antigen	312	273	53	LOC729603	Calcium binding
680	631	631	CCR6	Chemokine receptor 6	291	357	170	CREB1	cAMP response element-binding protein
648	648	521	AKR1D1	Aldo-keto reductase family 1, member D1	282	295	50	GJC1	Gap junction protein
599	580	548	LOC389517	Pseudogene	281	359	73	PDCD7	Promoter of apoptosis
529	463	180	FAM177A1		255	311	51	LOC642947	
485	504	466	PDE4C	Phosphodiesterase 4C	255	277	47	FKTN	Fukutin
475	570	537	RN7SL1	Cytoplasmic ribonucleoprotein complex	230	253	59	LILRB1	Leukocyte immunoglobulin-like receptor 1
453	399	337	AIRE	Involved in self tolerance and autoimmunity	200	251	47	SLC16A12	Solute carrier

Abbreviations: CON=control; CPM=Cyclophosphamide; OXP=oxaliplatin.

Spot-intensity signals from the CON samples were ranked according to their magnitudes, and those with values lower than that of the background/baseline (60) were defined as absent and excluded. There were just 92 genes passing these criteria, of which the top 40 are presented. The fates of the expressions of these genes in microvesicles from CPM- or OXP-treated samples were also assessed. Data were generated from two biological replicates.

**Table 2:** Identifying genes specific to OXP

		**Gene expression**				
**Symbol**	**Notes**	**CON**	**CPM**	**OXP**	**CPM/CON**	**OXP/CON**
*Falls*						
RPL7L1	Ribosomal protein	325	327	51	1.00	0.16
GJC1	Gap junction protein	282	295	50	1.04	0.18
FKTN	Fukutin	255	277	47	1.09	0.19
PNPT1	Polynucleotide phosphorylase 1	336	309	66	0.92	0.20
IL18	Supports NK action and stimulates the production of IFN*γ* by T-cells	330	304	76	0.92	0.23
LILRB1	Leukocyte immunoglobulin-like receptor 1	230	253	59	1.1	0.26
GABPB2	GA-binding protein transcription factor *β* subunit 2	395	371	194	0.94	0.49
						
*Rises*						
MAP4K4	MAPK member that has a role in the response to stress and cytokines	50	52	100	1.0	2.0
RHBDF2	Rhomboid-like protein	48	48	143	1.0	3.0
KRIT1	Ankyrin repeat-containing proteins involved with cytoskeletal processes	63	68	196	1.1	3.1
BCL7C	May be involved in anti-apoptotic responses	54	49	180	0.90	3.3
ABCA4	ATP-binding cassette	54	52	204	0.98	3.8
MAP1A	Microtubule protein involved with cytoskeletal re-arrangements	72	69	371	0.96	5.1

Abbreviations: BCL7C=B-Cell lymphoma 7C; CON=control; CPM=Cyclophosphamide; OXP=oxaliplatin.

Genes were filtered according to the criteria that expressions be (i) <10% different between untreated and CPM-treated, and (ii) >1-fold change in either direction between untreated and OXP-treated. This was done to generate a list of genes whose expressions were altered by treatment with OXP, but not by CPM. The ratios of expressions in CPM/control (CON) and OXP/CON are also shown. Data were generated from two biological replicates.
